# Impact of Selenium and Copper Nanoparticles on Yield, Antioxidant System, and Fruit Quality of Tomato Plants

**DOI:** 10.3390/plants8100355

**Published:** 2019-09-20

**Authors:** Hipólito Hernández-Hernández, Tomasa Quiterio-Gutiérrez, Gregorio Cadenas-Pliego, Hortensia Ortega-Ortiz, Alma Delia Hernández-Fuentes, Marcelino Cabrera de la Fuente, Jesús Valdés-Reyna, Antonio Juárez-Maldonado

**Affiliations:** 1 Instituto de Agroingeniería, Universidad del Papaloapan, Loma Bonita, Oaxaca 68400, Mexico; polo13_87-08@hotmail.com; 2 Maestría en Ciencias en Horticultura, Universidad Autónoma Agraria Antonio Narro, Saltillo, Coahuila 25315, Mexico; t.quiterio@hotmail.com; 3 Centro de Investigación en Química Aplicada, Saltillo, Coahuila 25294, Mexico; gregorio.cadenas@ciqa.edu.mx (G.C.-P.); hortensia.ortega@ciqa.edu.mx (H.O.-O.); 4 Instituto de Ciencias Agropecuarias, Universidad Autónoma del Estado de Hidalgo, Tulancingo, Hidalgo 43600, Mexico; hfad@hotmail.com; 5 Departamento de Horticultura, Universidad Autónoma Agraria Antonio Narro, Saltillo, Coahuila 25315, Mexico; marcelino.cabrera@uaaan.edu.mx; 6 Departamento de Botánica, Universidad Autónoma Agraria Antonio Narro, Saltillo, Coahuila 25315, Mexico; jesus.valdes@uaaan.edu.mx

**Keywords:** nanoparticles, Cu NPs, Se NPs, enzymatic compounds, antioxidants, chlorophyll, lycopene

## Abstract

The effects of nanoparticles (NPs) on plants are contrasting; these depend on the model plant, the synthesis of the nanoparticles (concentration, size, shape), and the forms of application (foliar, substrate, seeds). For this reason, the objective of this study was to report the impact of different concentrations of selenium (Se) and copper (Cu) NPs on yield, antioxidant capacity, and quality of tomato fruit. The different concentrations of Se and Cu NPs were applied to the substrate every 15 days (five applications). The yield was determined until day 102 after the transplant. Non-enzymatic and enzymatic antioxidant compounds were determined in the leaves and fruits as well as the fruit quality at harvest. The results indicate that tomato yield was increased by up to 21% with 10 mg L^−1^ of Se NPs. In leaves, Se and Cu NPs increased the content of chlorophyll, vitamin C, glutathione, 2,2′-azino-bis(3-ethylbenzthiazolin-6-sulfonic acid (ABTS), superoxide dismutase (SOD), glutathione peroxidase (GPX) and phenylalanine ammonia liasa (PAL). In fruits, they increased vitamin C, glutathione, flavonoids, firmness, total soluble solids, and titratable acidity. The combination of Se and Cu NPs at optimal concentrations could be a good alternative to improve tomato yield and quality, but more studies are needed to elucidate their effects more clearly.

## 1. Introduction

Selenium is not an essential element for plants, unlike humans and animals [1]. It is chemically similar to sulfur, which is why most plants cannot distinguish between one or the other, except for hyper-accumulating plants that prefer Se [2]. The soluble ways in which plants can absorb this element are selenate and selenite [3,4]. The absorption of this element is regulated by transporters that are found in the plasma membrane of the root. Selenate is transported by ion sulfate channels [5], while selenite is transported by phosphate transporters [6]. However, the route of application largely defines the accumulation of selenium in crops; it has been reported that the application via fertigation produces greater accumulation of selenium in the leaves of *Cichorium endivia* compared to the foliar application [7]. Several reports show that the application of a low concentration of selenium in plants stimulates the growth and the quality of fruits [8], improves photosynthesis [9], and activates the defense mechanism against abiotic [10] and biotic stresses [11]. Unlike selenium, copper is an essential element for plants that is involved in multiple functions such as electron transport during the process of photosynthesis and respiration, detection of ethylene, cell wall metabolism, protection against oxidative stress, and biogenesis of the molybdenum cofactor [12]. Copper is also widely used as a fungicide against certain pathogens. Both selenium and copper are contributed to crops in low concentrations, as they can cause toxicity in the plant.

With the advance of nanotechnology, the number of studies on nanoparticles (NPs) in crop plants has increased. NPs have different physicochemical properties compared to bulk materials. Several studies have shown that NPs applied at high concentrations (up to 2000 mg L^−1^) cause negative effects on morphology, physiology, and biochemistry [13,14] and also could cause genotoxicity in plants [15]. However, at appropriate concentrations, the NPs have positive effects. Particularly, selenium nanoparticles (Se NPs) have shown less toxicity in plants than selenate [16]. Selenate slows growth and decreases chlorophyll content through a disruption of the macroorganization of protein and pigment complexes in addition to a contraction of thylakoid membranes [17]. In contrast, selenium nanoparticles stimulate organogenesis and root growth [16]. This is because the uptake of Se NPs by the roots is slow, and then they oxidize rapidly inside the plant to selenite and become organic forms (selenocysteine and selenomethionine) [18]. Under short pulses of stress due to high and low temperatures, the Se NPs improved growth and relative water content in tomato plants [19]. On the other hand, copper nanoparticles (Cu NPs) are one of the most studied in relation to plants and have shown positive effects when used at low concentrations [20]. In tomato, Cu NPs induce numerous benefits, increase growth and yield, and improve enzymatic and non-enzymatic antioxidant systems and fruit quality [21,22,23,24,25].

As is well known, the tomato crop is one of the most important vegetables worldwide; in addition, tomato fruits are a source of antioxidants such as lycopene and β-carotene for the human diet. However, there is little research on the effects of Se NPs on plants [26] as well as their interaction with Cu NPs. Hence, the objective of this study was to evaluate the impact that different concentrations of Se NPs and Cu NPs have on the yield, the antioxidant capacity, and the fruit quality in tomato crops.

## 2. Results

### 2.1. Crop Yield

The Se and the Cu NPs showed significant differences in the variables of average fruit weight and yield (Table 1). The interaction of 20 mg L^−1^ of Se NPs and 10 mg L^−1^ of Cu NPs increased the average weight of tomato fruit by 25% compared to the control. Likewise, with the concentration of 10 mg L^−1^ of Se NPs, the yield per plant was increased up to 21%. Although there were no differences in the number of fruits per plant, this treatment was the one that presented the highest number, which could influence the highest yield. None of the concentrations of Se or Cu NPs negatively affected the fruit yield of tomato plants. 

### 2.2. Chlorophyll Content in Leaves

The Se and the Cu NPs showed significant effects on the chlorophyll content in tomato leaves (Table 2). When applied individually, the NPs induced a decrease in these pigments—the Se NPs (1 and 10 mg L^−1^) and the Cu (10, 50 and 250 mg L^−1^) decreased the chlorophyll content of the leaves. In contrast, the interaction of 1 mg L^−1^ of Se NPs and 50 mg L^−1^ of Cu NPs increased the chlorophyll content a, b, and total (20, 17, and 19%, respectively). In addition, it was observed that the interaction of Se NPs at 1 mg L^−1^ in combination with the three doses of Cu NPs increased the chlorophyll a/b ratio, while the rest of the treatments generally decreased this relationship.

### 2.3. Non-Enzymatic Antioxidant Compounds in Leaves

Interaction of Se and Cu NPs significantly modified the non-enzymatic compounds of tomato leaves (Table 3 and Table 4). The interaction of 1 mg L^−1^ of Se NPs and 10 mg L^−1^ of Cu NPs increased vitamin C content up to 32%. The interaction of 1 mg L^−1^ of Se NPs with Cu NPs in all concentrations increased the concentration of glutathione in leaves. Also, the application of Se NPs at 20 and 10 mg L^−1^ improved the content of vitamin C and glutathione in leaves by 21 and 27%, respectively. In contrast, the interactions of 1 mg L^−1^ of Se NPs with 10 and 50 mg L^−1^ of Cu NPs decreased the flavonoid content in leaves (14 and 16%, respectively). None of the concentrations of Se and Cu NPs significantly modified the total phenolic content with respect to the control (Table 3).

The interaction of Se NPs at 10 mg L^−1^ with all concentrations of Cu NPs increased the 2,2′-azino-bis(3-ethylbenzthiazolin-6-sulfonic acid (ABTS) antioxidant capacity of the hydrophilic compounds in leaves (Table 4). The interaction of Se NPs (20 mg L^−1^) and Cu NPs (250 mg L^−1^) increased the hydrophilic and the lipophilic ABTS antioxidant capacity in leaves (14 and 9%, respectively). The interaction of Se NPs (20 mg L^−1^) with 50 and 250 mg L^−1^ of Cu NPs increased the ABTS total antioxidant capacity in leaves (5.8 and 12% respectively). 

### 2.4. Enzymatic Activity in Leaves

The Se and the Cu NPs significantly modified the enzymatic activity [ascorbate peroxidase (APX), glutathione peroxidase (GPX), superoxide dismutase (SOD), and phenylalanine ammonia liasa (PAL)] in tomato leaves; only in the catalase enzyme, no effect was observed (Table 5). The interaction of Se NPs at 20 mg L^−1^ with Cu NPs at 50 mg L^−1^ increased the activity of the APX enzyme (159%). Se NPs at 1 mg L^−1^ with Cu NPs at 10 and 50 mg L^−1^ increased the activity of the GPX enzyme (1726 and 2600%, respectively). The application of 1 mg L^−1^ of Se NPs alone increased the SOD enzyme activity up to 63%. Se NPs at 1 mg L^−1^ and Cu NPs at 10 mg L^−1^ increased the PAL enzyme activity up to 203%. In addition, 50 mg L^−1^ of Cu NPs also increased the activity of this enzyme up to 147%.

### 2.5. Non-Enzymatic Antioxidant Compounds in Fruits

The Se and the Cu NPs significantly modified the non-enzymatic antioxidant compounds of tomato fruits (Table 6). The interaction of Se NPs at 1 mg L^−1^ and Cu NPs at 50 mg L^−1^ increased vitamin C content up to 36%, and 10 mg L^−1^ of Se NPs with 250 mg L^−1^ of Cu NPs generated the highest glutathione content, being 38% more than the control. In addition, the application of 10 mg L^−1^ of Se NPs also induced an increase in glutathione content (25%) compared to the control, and this treatment in combination with 50 mg L^−1^ of Cu NPs induced an increase of 24%. The concentration of 20 mg L^−1^ of Se NPs alone and in combination with 10 mg L^−1^ of Cu NPs increased the flavonoid content (26% and 29%, respectively). In contrast, the interaction of Se NPs at 10 mg L^−1^ and Cu NPs at 10 mg L^−1^ as well as all concentrations of Cu NPs decreased the lycopene content of tomato fruits. Regarding the content of total phenols and β-carotene in fruits, none of the concentrations of Se and Cu NPs modified these compounds.

### 2.6. Fruit Quality

Se and Cu NPs significantly modified tomato fruit quality, especially firmness, total soluble solids, and titratable acidity (Table 7). The interaction of 10 mg L^−1^ of Se NPs and 50 mg L^−1^ of Cu NPs increased the firmness of the fruits by 48% compared to the control. The interaction of 20 mg L^−1^ of Se NPs and 250 mg L^−1^ of Cu NPs increased the total soluble solids content in tomato fruits (10%). The concentration of 20 mg L^−1^ of Se NPs with 10 or 50 mg L^−1^ of Cu NPs increased the titratable acidity of the fruits up to 20% with respect to the control, as did 10 mg L^−1^ of Se NPs with 250 mg L^−1^ of Cu NPs.

## 3. Discussion

NPs can interact with plant cells at a physicochemical level that is independent of the type of material used, since they depend more on the surface properties [27]. For this reason, it is possible to find in the literature a variety of responses ranging from negative effects on crops [13,14] to positive effects at the level of growth, yield, and antioxidant defense systems, among others [21,22,23,24,25]. Additionally, NPs can provide essential or beneficial elements to the plant, causing the typical responses of each element [27]—for example, the stimulation of the growth and the quality of fruits by the application of selenium [8] or the multiple functions performed by the Cu as an essential element [12]. Another important issue that must be taken into account is the route of application of NPs, as this can directly influence plant responses. In this work, the application was via soil, and although the stability of the NPs in the soil was not analyzed, it is important to mention that there are several chemical and biological factors that determine the transformation of NPs and therefore their bioavailability and their translocation of the soil to the plant [28]. However, in the present work, it was shown that the application of Se and Cu NPs induced positive responses in tomato plants, which is consistent with that reported in the literature.

Previous studies showed that 10 mg L^−1^ of Cu NPs increased tomato growth and yield [21,22]. Likewise, in plants of *Cyamopsis tetragonoloba,* it was shown that the Se NPs increased the yield [29]. This could be due to the fact that Se NPs stimulate the formation of organs in plants [16,27]. In corn, zinc (Zn) NPs (0.16%) increased yield by up to 40% [30]. Likewise, Zn NPs (25 mg L^−1^) increased yield in *Phaseolus vulgaris* L. [31]. Therefore, Se and Cu NPs could be a good alternative to improve the yield of tomato plants.

Zsiros et al. [17] reported that Se NPs (100 mg L^−1^) decreased the chlorophyll content of tobacco leaves without affecting the structure and the function of photosynthetic machinery; this was due to the limited penetration into the leaf tissue. This decrease in chlorophyll content was due to the oxidative stress caused by the Se NPs, which caused a peroxidation of the chloroplast membrane [32]. Da Costa and Sharma [14] reported that CuO NPs (100 and 1000 mg L^−1^) decreased the content of photosynthetic pigments in rice leaves, which was reflected in the decrease of photosynthesis. Chung et al. [33] reported that, in *Brassica rapa,* the CuO NPs (50, 250, and 500 mg L^−1^) decreased the chlorophyll content. Another study on the interaction between CuO NPs and chlorophyll extracted from wheat leaves using fluorescence spectroscopy reported that the life of chlorophyll decreased as the concentration of CuO NPs increased [34]. In contrast, Hussein et al. [32] reported that Se NPs (20 mg L^−1^) increased the chlorophyll content in peanut leaves due to the protection that Se NPs provided over photosynthetic pigments. This type of response depends on the dose used, since NPs can induce positive responses to certain doses, while in others, they can induce the opposite effect or simply have no effect. This behavior, called hormesis, has been reported when NPs are applied as biostimulants in crops [27]. Therefore, the combination of Se and Cu NPs could improve the chlorophyll content in tomato leaves at adequate concentrations.

The enzymatic and the non-enzymatic antioxidant systems (ascorbate, glutathione, phenols, flavonoids, etc.) maintain the balance of reactive oxygen species in different plant cellular reactions. Ascorbate (vitamin C) is the most abundant antioxidant in plants and is used as a cofactor for redox enzymes. It is synthesized in the mitochondria, and then it is transported to the chloroplasts by means of a phosphate transporter (*AtPHT4; 4*). In the chloroplast, it serves to dissipate energy in the form of heat and eliminate free radicals during the process of photosynthesis [35]. The most important function of ascorbate in plant cells is the donation of electrons (ascorbate-glutathione cycle), where two ascorbate molecules are used by the enzyme ascorbate peroxidase (APX) to reduce H_2_O_2_ to water and monodehydroascorbate (MDA) [36]. This in turn can be recycled through the enzyme dehydroascorbate reductase [37]. This study showed that the Se and the Cu NPs increased the amount of ascorbate from tomato leaves; this could have been due to the stimulation of NPs [27] and to a greater activity of the enzyme dehydroascorbate reductase [37]. Also, the increase in ascorbate in tomato leaves induced by Se and Cu NPs could make the photosynthetic apparatus efficient by protecting the complexes of light collection induced by high radiation (xanthophyll cycle) [38]. 

Glutathione has important functions in the development of plants that cannot be performed by other thiols or antioxidants—it interacts with various proteins through the exchange of thiol-disulfide. Some functions include biosynthetic pathways, detoxification, antioxidant biochemistry, and redox homeostasis [39]. Glutathione is synthesized from constituent amino acids such as cysteine in different cell compartments and moves through the apoplast or the symplast [39]. It acts as a reducing compound of reactive oxygen species and cellular reducers and also fulfills signaling functions. In the ascorbate-glutathione cycle, it is used to reduce dehydroascorbate, both enzymatic and non-enzymatic, and is also oxidized to oxidized glutathione (GSSG); to regenerate, it is catalyzed by the enzyme glutathione reductase and nicotinamide-adenine-dinucleotide-phosphate (NADPH) as reducing power [40]. This study shows that the Se and the Cu NPs stimulate the increase of glutathione in tomato leaves, which could be due to the increase in sulfur assimilation and to a greater enzymatic activity of glutathione reductase [39]. 

Flavonoids are secondary metabolites that have regulatory functions in plant development, UV protection, and defense and signaling mechanisms [41]. In addition, when replaced by the dihydroxy B-ring, they act as antioxidants in plants. They are synthesized in the endoplasmic reticulum and then transported to the chloroplast to eliminate singlet oxygen, or they are present in the nucleus of the mesophilic cells and act as reactive oxygen species (ROS) inhibitors that complex with iron (Fe) and Cu ions [42]. Vacuolar dihydroxy B-ring serves as a co-substrate for vacuolar peroxidases to reduce the H_2_O_2_ of chloroplast after depletion of ascorbate [42]. Cumplido-Nájera et al. [43] reported that Cu NPs decreased the flavonoid content in tomato leaves. In contrast to our results, Hussein et al. [32] reported that Se NPs (40 mg L^−1^) increased flavonoid content in peanut plants. Therefore, the Se and the Cu NPs could modify the flavonoid content in tomato leaves, which could modify the signaling and the response mechanisms.

Reactions with the ABTS cation involve an electron transfer process. In the test, the ABTS generated with the oxidation of K_2_S_2_O_8_ is discolored when it reacts with antioxidants that donate hydrogen [44]. This test is used to measure antioxidant capacity. It has been shown that Se NPs have the ability to eliminate ABTS radicals [45]. Other NPs based on cerium and titanium have shown the ability to eliminate free radicals in plants [46,47]. Therefore, adequate concentrations of Se and Cu NPs increase the antioxidant capacity in tomato leaves. Here, we show that Se and Cu NPs activate the non-enzymatic antioxidant system of tomato plants through an improvement in ascorbate-glutathione and the ability to eliminate reactive oxygen species. This may be due to the interaction between the surface charges of the NPs and the cell membrane causing a biostimulant effect on enzymatic and non-enzymatic compounds [27].

Selenium and copper act as catalytic centers of proteins in the cellular metabolism of plants [3,48] and regulate the activities of enzymes such as SOD, catalase (CAT), APX, and GPX that are important ROS eliminators and form the first line of defense against oxidative stress. Hussein et al. [32] reported that Se NPs (20–40 mg L^−1^) increased the activity of APX and peroxidase (POX) enzymes and decreased CAT in peanut plants. Also, Cu NPs (10 mg L^−1^) increased the activity of SOD, CAT, APX, and GPX enzymes in tomato leaves [22]. Therefore, Se and Cu NPs could improve the enzymatic antioxidant defense mechanism of tomato plants. Additionally, Se and Cu NPs activated the PAL enzyme in tomato leaves. It was previously shown that Cu NPs increase the activity of this enzyme [22,43]. Selenium is also considered an important activator of the PAL enzyme in plants [49]; therefore, the results observed are consistent with those reported in the literature. The PAL enzyme is the main precursor to the synthesis of phenylpropanoids in plants that act as inhibitors of singlet oxygen formation, free radical scavengers, and reducing agents against abiotic [50] and biotic stress [43]. In this way, the plants can be prepared to cope with some kind of stress, whether biotic or abiotic.

It has been shown that Cu NPs [23,25] and selenium [51] increase antioxidant compounds in tomato fruits. Similarly, in this study, we showed that the Se and the Cu NPs increased the content of vitamin C, glutathione, and flavonoids in tomato fruits. During the early stages of ripening of the tomato fruit (green-yellow), the antioxidant enzyme system protects the fruit from oxidative damage, and as the ripening progresses (ripe red), they decrease [52]. Ascorbate and glutathione also decrease as fruit ripening progresses [53]. This decrease is due to the appearance of carotenoids (lycopene and β-carotene), mainly in the stage of maturation breaker [54]. In this study, the carotenoids (lycopene) were decreased by the NPs; this could have been linked to the NPs increasing the concentration of ascorbate and glutathione, reflecting a delay in fruit ripening. In tomato fruits, it has been possible to improve the content of carotenoids and flavonoids simultaneously through genetic engineering; otherwise, it is only possible to increase carotenoids or flavonoids, but not both [55]. Here, we showed that Se and Cu NPs increased flavonoid content but decreased lycopene. These antioxidant compounds are important in the human diet. Due to the inability for humans to synthesize vitamin C, the main source is fruits [56]. A diet rich in flavonoids is associated with a lower risk of cancer diseases and prevention of many diseases [57], hence the importance of increasing the concentration of antioxidant compounds in fruits from pre-harvest management.

Zhu et al. [51] mention that selenium could be a good strategy to retain ripening and increase the shelf life of tomato fruits. In this study, the increase in firmness in fruits caused by the NPs could have been due to the lignification of the pericarp cell wall [25]. This result was related to the increased activity of the PAL enzyme; phenylalanine is a precursor to lignin synthesis [58]. With the increase of firmness of fruits, the shelf life could be prolonged. Naturally, climacteric fruits such as tomatoes increase the sugar content and decrease organic acids as the ripening stages progress [59]. Sugars (sucrose, glucose, and fructose) are imported as photosynthates, while organic acids (citrate and malate) are synthesized from sugars imported from glycolysis that regulate starch and cell wall degradation [60]. Starch determines an important role in the accumulation of soluble solids in ripe fruit [61]. Therefore, the results obtained here suggest that Se and Cu NPs can increase the shelf life of tomato fruits and also could modify soluble solids and citric acid of tomato fruits by improving the quality of the fruit. This could have interesting applications in managing the quality of tomato fruits from a commercial point of view.

## 4. Materials and Methods 

### 4.1. Crop Development and Management

In a multi-tunnel type greenhouse with polyethylene cover, saladette tomato plants “El Cid F1” (Harris Moran, Davis, CA, USA) of indeterminate growth were established under a soilless cultivation system. The transplant was carried out in black polyethylene bags of 10 L capacity using a mixture of peat moss and perlite as a substrate in 1:1 (v/v) ratio. Pruning was used on one stem, and the crop was developed until 102 days after transplantation (dat). The application of nutrients was through an irrigation system directed using Steiner nutrient solution [62].

### 4.2. Treatments

The treatments used were three concentrations of Se NPs (1, 10, and 20 mg L^−1^) and Cu NPs (10, 50, and 250 mg L^−1^) plus one control. From 11 days after transplantation, five applications of each of the NPs concentrations were made directly to the substrate in two week intervals. Each of the applications consisted of 2.5, 5.5, 13, 17.1, and 17.1 mL per plant at the concentrations of each treatment during the experiment. Se NPs and Cu NPs were synthesized at the Applied Chemistry Research Center (Saltillo, Mexico) according to the methodologies of Quiterio-Gutiérrez [63] and Cadenas-Pliego [64], respectively. The Se NPs were 2–20 nm in size [63], and the Cu NPs were 42 nm [43], and both were spherical in shape.

### 4.3. Sampling of Leaves and Fruits

Random plants were selected, and four fully expanded young leaves were sampled. At harvest stage, 12 fruits of uniform size and red in the ripening stage 6 were selected according to the United States Department of Agriculture (USDA) scale [65]. Half of the sampled leaves and fruits were used for fresh determinations. The other half was stored at −20 °C and then lyophilized for 72 h at −84 °C and 0.060 mbar (Labconco, FreeZone 2.5 L model, Kansas City, MO, USA). 

### 4.4. Photosynthetic Pigments

The content of chlorophyll [mg 100 g^−1^ fresh weight (FW)] was determined using the method of Nagata and Yamashita [66]. For this, the absorbances at 645 and 663 nm were determined and used in Equations (1) and (2). Total chlorophyll (Chl) was considered to be the sum of Chl a and Chl b.

Chla = 0.999 × Abs_663_ − 0.0989 × Abs_645_ (1)

Chlb = −0.328 × Abs_663_ + 1.77 × Abs_645_ (2)

### 4.5. Non-Enzymatic Antioxidants

Lycopene and β-carotene [mg 100 g^−1^ dry weight (DW)] were determined according to Nagata and Yamashita [66] using the absorbance values of 453, 505, 645, and 663 nm in Equations (3) and (4).

Lycopene = −0.0458 × Abs_663_ + 0.204 × Abs_645_ + 0.372 × Abs_505_ − 0.0806 × Abs_453_ (3)

β-carotene = 0.216 × Abs_663_ − 1.22 ×Abs_645_ − 0.304 × Abs_505_ + 0.452 × Abs_453_ (4)

Vitamin C (mg 100 g^−1^ FW) was determined by the colorimetric method using 2,6 dichlorophenol, 1 g of fresh tissue, and HCl (2%), as described in Padayatt et al. [67].

Glutathione (mg 100 g^−1^ DW) was determined using the method of Xue et al. [68] by means of a 5,5-dithio-bis-2 nitrobenzoic acid (DTNB) reaction. A mix of 0.480 mL of the extract, 2.2 mL of sodium dibasic phosphate (Na_2_HPO_4_ at 0.32 M), and 0.32 mL of the DTNB dye (1 mM) was placed in a test tube. Then, the mix was vortexed and read on a UV-Vis spectrophotometer (Thermo Fisher Scientific, G10S model, Waltham, MA, USA) at 412 nm using a quartz cell.

Flavonoids (mg 100 g^−1^ DW) were determined by the method of Arvouet-Grand et al. [69]. For the extraction, 100 mg of lyophilized tissue was placed in a test tube, where 10 mL of reagent grade methanol was added and shaken for 30 s until the mixture was homogenized. The mixture was filtered using No. 1 Whatman paper. For the quantification, 2 mL of the extract and 2 mL of methanolic solution of aluminum trichloride (AlCl_3_) 2% were added to a test tube and left to rest for 20 min in the dark. The reading was then taken in a UV-Vis spectrophotometer (Thermo Fisher Scientific, G10S model, Waltham, MA, USA) at a wavelength of 415 nm using a quartz cell.

Phenols (mg 100 g^−1^ DW) were determined with Folin–Ciocalteu reagent, as described in Cumplido-Nájera et al. [43]. The sample (0.2 g) was extracted with 1 mL of a water:acetone solution (1:1). The mixture was vortexed for 30 s. The tubes were centrifuged (Thermo Scientific Mod. ST 16R centrifuge, Langenselbold, Germany) at 17,500× g for 10 min at 4 °C. In a test tube, 50 μL of the supernatant, 200 μL of the Folin–Ciocalteu reagent, 500 μL of 20% sodium carbonate (Na_2_CO_3_), and 5 mL of distilled water were added and then vortexed for 30 s. The samples were placed in a water bath at 45 °C for 30 min. Finally, the reading was taken at an absorbance of 750 nm using a plastic cell in a UV-Vis spectrophotometer (Thermo Fisher Scientific, G10S model, Waltham, MA, USA).

The antioxidant activity by ABTS was determined by the spectrophotometric method of Re et al. [70], which is based on the discoloration of the ABTS radical cation. This radical was obtained from the reaction of ABTS at 7 mM with potassium persulfate at 2.45 mM (1:1) in the dark at 26 °C for 16 h and then diluted with 20% ethanol to obtain an absorbance of 0.7 ± 0.01 at 750 nm. Afterwards, to determine antioxidant capacity in the hydrophilic compounds, phosphate buffer, 5 μL of extract, and 245 μL of the ABTS radical dilution (7 mM) were placed in a microplate and stirred for 5 s and then allowed to stand for 7 min in darkness. The absorbance was measured by a plate reader (BioTek, ELx808 model, Winooski, VT, USA) at a wavelength of 750 nm. The blank was prepared with 250 μL of phosphate buffer (pH 7.0–7.2, 0.1 M). For the determination of the same in lipophilic compounds, extraction was carried out with a hexane:acetone solution. The results are expressed as Trolox equivalents in µmol per gram of dry weight (µmol g^−1^ DW).

### 4.6. Enzymatic Activity

The quantification of total proteins (mg g^−1^ of DW) was performed using Bradford’s colorimetric technique [71]. In a microplate, 5 μL of the extract and 250 μL of Bradford reagent were placed in each well. They were incubated for 10 min at room temperature (26 °C) and then read at a wavelength of 630 nm on a microplate reader (BioTek, ELx808 model, Winooski, VT, USA).

Catalase (CAT) (EC 1.11.1.6) (U TP^−1^, where U is equal to the mM equivalent of H_2_O_2_ consumed per milliliter per minute) was quantified by the method of Dhindsa et al. [72]. The measurement was carried out in two steps [at time 0 (T0) and at time 1 (T1)]. At T0, 100 μL of extract, 400 μL of H_2_SO_4_ (5%), and 1 mL of H_2_O_2_ (100 mM) were added to an Eppendorf tube and vortexed for 30 s. The absorbance was then measured on a UV-Vis spectrophotometer (Thermo Fisher Scientific, G10S model, Waltham, MA, USA) with a quartz cell at 270 nm. At T1, 100 μL of extract and 1 mL of H_2_O_2_ (100 μL) were added and stirred for 1 min in a vortex at 26 °C. Then, 400 μL of H_2_SO_4_ (5%) was added to stop the reaction, and the absorbance was measured by a UV-Vis spectrophotometer (Thermo Fisher Scientific, G10S model, Waltham, MA, USA) with a quartz cell at 270 nm. The determination of catalase was based on the quantification of the oxidation rate of H_2_O_2_ by absorbance difference (T0–T1).

Superoxide dismutase (SOD) (EC 1.15.1.1) (U mL^−1^, where U is defined as the amount of enzyme needed to exhibit 50% dismutation of the superoxide radical) was carried out using the SOD Cayman 706002^®^ kit. A mix of 20 μL of extract, 200 μL of the radical detector (tetrazolium salt), and 20 μL of xanthine oxidase solution was placed in a microplate. The microplate was covered with a transparent cover (kit), stirred for 10 s, and then incubated at 26 °C for 30 min. The absorbance was then measured at a length of 450 nm using a plate reader (BioTek, ELx808 model, Winooski, VT, USA). The principle of the test was based on the use of a tetrazolium salt for the detection of superoxide radicals generated by xanthine oxidase and hypoxanthine.

Glutathione peroxidase (GPX) (EC 1.11.1.9) [U per gram of total proteins (U TP^−1^), where U is equal to the mM equivalent of reduced glutathione (GSH) per milliliter per minute] was determined by the method of Flohé and Günzler [73]. A mix of 200 μL of extract, 400 μL of GSH (0.1 mM), and 200 μL of Na_2_HPO_4_ (0.067 M) was placed in a test tube. The mixture was preheated in a water bath at 25 °C for 5 min, then 200 μL of H_2_O_2_ (1.3 mM) was added to start the catalytic reaction for 10 min at a temperature of 26 °C. The reaction was stopped by the addition of 1 mL of 1% trichloroacetic acid. The mixture was placed in an ice bath for 30 min and then centrifuged at 1008× *g* for 10 min at 4 °C. To assess the glutathione peroxidase, 480 μL of the supernatant, 2.2 mL of Na_2_HPO_4_ (0.32 M), and 320 μL of 5,5-dithio-bis-2-nitrobenzoic acid dye (DTNB) of 1 mM were placed in a test tube. The absorbance was measured by a UV-Vis spectrophotometer (Thermo Fisher Scientific, G10S model, Waltham, MA, USA) at 412 nm with a quartz cell.

Ascorbate peroxidase (APX) (EC 1.11.1.1) was determined by the method of Nakano and Asada [74] and is expressed as U per gram of total proteins (U g^−1^ TP), where U is equal to the μmol of oxidized ascorbate per milliliter per minute. The measurement was undertaken at two moments [at time 0 (T0) and at time 1 (T1)]. At T0, a mix of 100 μL of extract, 500 μL of ascorbate (10 mg L^−1^), 400 μL of H_2_SO_4_ (5%), and 1 mL of H_2_O_2_ (100 mM) were placed in a test tube and then vortexed for 30 s. The absorbance was measured in a UV-Vis spectrophotometer (Thermo Fisher Scientific, G10S model, Waltham, MA, USA) at 266 nm with a quartz cell. At T1, 100 μL of extract, 500 μL of ascorbate (10 mg L^−1^), and 1 mL of H_2_O_2_ (100 mM) were added to the previous mixture and vortexed for 1 min at a temperature of 26 °C. To stop the reaction, 400 μL of H_2_SO_4_ (5%) was added, and the absorbance was measured. Ascorbate peroxidase determination was based on the quantification of the ascorbate oxidation rate by means of the absorbance difference (T0–T1).

The activity of phenylalanine ammonia lyase (PAL) (EC 4.3.1.5) was determined according to Sykłowska-Baranek et al. [75], and the results expressed as U per gram of total proteins (U g^−1^ TP), where U is equal to µmol equivalent of transcinnamic acid per milliliter per minute. A total of 0.1 mL of the enzymatic extract was taken, and 0.9 mL of L-phenylalanine (6 mM) was added. After 30 min of incubation at 40 °C, the reaction was stopped with 0.25 mL of 5 N HCl. The samples were placed in an ice bath, and 5 mL of distilled water was added. The absorbance was determined at 290 nm on a UV-Vis spectrophotometer (Thermo Fisher Scientific, G10S model, Waltham, MA, USA).

### 4.7. Fruit Quality

The parameters that describe a fruit’s quality [hydrogen potential (pH), total soluble solids (TSS), fruit firmness, and titratable acidity (TA)] were determined as described in López-Vargas et al. [25]. For this, six fruits (one per plant) of uniform size and in a light red state of maturity were collected from the third cluster.

### 4.8. Statistical Analysis

Six replicates per treatment were considered for each of the evaluated variables in a factorial experiment (4*4) in a completely random design. Each replicate was obtained from one different plant. The analysis of variance and Fisher least significant difference (LSD) mean test (*p* *≤* 0.05) were applied to each variable. All statistical processes were performed using the software Infostat 2018 (http://www.infostat.com.ar). 

## 5. Conclusions

The beneficial effects of Se and Cu NPs on tomato plants depend on the concentration used. Se NPs improve the yield of tomato plants, while in conjunction with Cu NPs, they do not induce greater yield—in fact, in certain doses, they can decrease it.

The application of Se and Cu NPs increases the chlorophyll content and improves the enzymatic (SOD, CAT, GPX, and PAL) and the non-enzymatic antioxidant system (vitamin C and glutathione) in the leaves of tomato plants. In fruits, they also induce positive effects, increase non-enzymatic antioxidant compounds (vitamin C, glutathione and flavonoids), firmness, total soluble solids, and titratable acidity, which improve nutraceutical and commercial quality.

The Se and the Cu NPs could be a good alternative to improve tomato crop productivity, especially for the production of higher quality crops. However, more research is needed to clarify the effects of Se and Cu NPs on crop plants.

## Figures and Tables

**Table 1:** Tomato crop yield with application of selenium (Se) and copper (Cu) nanoparticles (NPs).

Se NPs (mg L^−1^)	Cu NPs (mg L^−1^)			
	0	10	50	250
**^NS^ Fruit number**				
**^NS^ 0**	^NS^ 64.67 ± 2.9 a	69.17 ± 4.3 a	65.28 ± 3.3 a	67.22 ± 4.7 a
**1**	64.78 ± 5.9 a	62.89 ± 4.4 a	65.22 ± 3.3 a	70.56 ± 3.3 a
**10**	73.61 ± 4.4 a	63.22 ± 1.9 a	66.67 ± 2.5 a	66.56 ± 4.6 a
**20**	63.11 ± 3.0 a	61.78 ± 4.2 a	64.94 ± 6.4 a	63.89 ± 4.6 a
**^NS^ Average Fruit Weight (g)**				
**^NS^ 0**	* 90.26 ± 3.5 bc	87.71 ± 4.9 bc	96.43 ± 4.4 abc	85.06 ± 5.5 c
**1**	93.93 ± 2.9 bc	102.50 ± 4.7 ab	99.11 ± 4.2 abc	87.41 ± 5.4 bc
**10**	94.98 ± 2.4 bc	85.01 ± 9.0 c	98.32 ± 4.2 abc	97.56 ± 5.2 abc
**20**	87.81 ± 4.8 bc	112.66 ± 14.8 a	85.74 ± 3.5 c	90.41 ± 6.1 bc
**^NS^ Yielg (g plant^−1^)**				
**^NS^ 0**	* 5807.49 ± 274 bc	6014.29 ± 454 abc	6327.44 ± 475 abc	5609.54 ± 355 c
**1**	5982.36 ± 431 abc	6319.41 ± 279 abc	6373.23 ± 202 abc	6154.06 ± 502 abc
**10**	7001.19 ± 476 a	5443.59 ± 651 c	6514.69 ± 257 abc	6324.67 ± 219 abc
**20**	5503.52 ± 340 c	6849.89 ± 917 ab	5551.78 ± 573 c	5806.49 ± 555 bc

ANOVA significance for both main factors and interactions are ^NS^ (not significant), and * <0.05. Means ± standard error between rows and columns with a common letter are not significantly different according to Fisher´s least significant difference test (*p* ≤ 0.05).

**Table 2:** Chlorophyll content in tomato leaves from plants treated with Se and Cu NPs.

Se NPs (mg L^−1^)	Cu NPs (mg L^−1^)			
	0	10	50	250
**^NS^ Chlorophyll a (mg 100 g^−1^ FW)**				
*** 0	*** 81.38 ± 3.5 c	64.51 ± 2.6 de	43.80 ± 1.5 gh	48.17 ± 1.5 fg
**1**	49.55 ± 0.8 fg	92.68 ± 3.2 ab	97.83 ± 1.9 a	85.58 ± 8.3 bc
**10**	41.65 ± 1.9 gh	61.41 ± 1.7 e	52.52 ± 1.4 f	79.06 ± 1.2 c
**20**	82.48 ± 2.0 c	35.83 ± 1.7 h	70.28 ± 1.9 d	42.61 ± 1.6 gh
* Chlorophyll b (mg 100 g^−1^ FW)				
*** 0	*** 35.08 ± 1.6 b	28.43 ± 1.2 cd	19.88 ± 0.7 h	20.67 ± 0.6 gh
**1**	24.39 ± 0.5 ef	37.68 ± 1.3 b	41.12 ± 0.8 a	35.38 ± 3.4 b
**10**	19.48 ± 0.9 h	27.13 ± 0.8 de	23.56 ± 0.7 fg	35.61 ± 0.5 b
**20**	37.38 ± 0.8 b	15.84 ± 0.7 i	31.34 ± 0.9 c	19.05 ± 0.7 hi
**^NS^ Total Chlorophyll (mg 100 g^−1^ FW)**				
*** 0	*** 116.46 ± 5.1 c	92.94 ± 3.7 de	63.68 ± 2.1 gh	68.84 ± 2.2 fgh
**1**	73.94 ± 1.3 fg	130.36 ± 4.4 ab	138.95 ± 2.7 a	120.96 ± 11.7 bc
**10**	61.13 ± 2.8 hi	88.54 ± 2.5 e	76.08 ± 2.0 f	114.67 ± 1.7 c
**20**	119.86 ± 2.8 bc	51.66 ± 2.5 i	101.62 ± 2.7 d	61.66 ± 2.3 hi
*** Chlorophyll a/b ratio				
*** 0	*** 2.32 ± 0.01 d	2.27 ± 0.01 e	2.20 ± 0.01 h	2.33 ± 0.01 d
**1**	2.03 ± 0.01 j	2.46 ± 0.01 a	2.38 ± 0.01 c	2.42 ± 0.01 b
**10**	2.14 ± 0.01 i	2.26 ± 0.01 ef	2.23 ± 0.01 g	2.22 ± 0.01 gh
**20**	2.21 ± 0.01 h	2.26 ± 0.01 ef	2.24 ± 0.01 fg	2.24 ± 0.01 fg

ANOVA significance for both main factors and interactions are ^NS^ (not significant), * <0.05, **< 0.01, ***< 0.001. Means ± standard error between rows and columns with a common letter are not significantly different according to Fisher´s least significant difference test (*p* ≤ 0.05). FW: fresh weight.

**Table 3:** Non-enzymatic antioxidant compounds in tomato leaves from plants treated with Se and Cu NPs.

Se NPs (mg L^−1^)	Cu NPs (mg L^−1^)			
	0	10	50	250
**^NS^** **Vitamin C (mg 100 g^−1^ FW)**				
*** 0	*** 10.42 ± 0.8 cde	10.12 ± 0.5 de	10.87 ± 0.5 cde	9.83 ± 0.6 de
**1**	9.70 ± 0.3 de	13.78 ± 0.9 a	11.75 ± 0.6 bc	11.28 ± 0.7 bcd
**10**	9.83 ± 0.5 de	9.38 ± 0.5 e	9.55 ± 0.4 e	9.40 ± 0.3 e
**20**	12.60 ± 0.4 ab	10.87 ± 0.5 cde	9.53 ± 0.5 e	10.43 ± 0.5 cde
**^NS^ Glutathione (mg 100 g^−1^ DW)**				
** 0	* 6.97 ± 0.3 d	7.55 ± 0.3 bcd	7.45 ± 0.3 bcd	7.22 ± 0.4 cd
**1**	7.82 ± 0.6 abcd	8.16 ± 0.4 abc	8.88 ± 0.4 a	8.81 ± 0.3 a
**10**	8.83 ± 0.4 a	8.43 ± 0.6 ab	7.93 ± 0.4 abcd	7.76 ± 0.6 abcd
**20**	8.12 ± 0.3 abcd	7.53 ± 0.4 bcd	8.20 ± 0.4 abc	8.08 ± 0.4 abcd
**^NS^ Flavonoids (mg 100 g^−1^ DW)**				
*** 0	* 301.77 ± 20.0 abc	265.47 ± 13.3 cde	314.58 ± 19.0 ab	326.91 ± 16.2 a
**1**	271.87 ± 11.5 cde	259.93 ± 19.4 de	252.47 ± 14.6 e	276.93 ± 6.4 bcde
**10**	333.21 ± 19.4 a	300.06 ± 12.6 abcd	272.82 ± 10.8 cde	274.64 ± 7.5 bcde
**20**	265.47 ± 13.7 cde	245.50 ± 11.7 e	281.04 ± 12.5 bcde	266.80 ± 17.6 cde
**^NS^ Phenols (mg 100 g^−1^ DW)**				
* 0	* 481.42 ± 16.4 abcde	517.27 ± 15.8 abc	494.83 ± 17.2 abc	410.90 ± 32.6 e
**1**	412.36 ± 26.3 de	450.82 ± 24.3 bcde	466.56 ± 19.8 bcde	525.57 ± 25.5 ab
**10**	448.93 ± 17.5 cde	468.02 ± 24.3 bcde	498.32 ± 27.16 abc	487.11 ± 42.1 abcde
**20**	514.50 ± 12.5 abc	548.45 ± 15.1 a	487.54 ± 60.2 abcd	506.49 ± 12.9 abc

ANOVA significance for both main factors and interactions are ^NS^ (not significant), * <0.05, ** <0.01, *** <0.001. Means ± standard error between rows and columns with a common letter are not significantly different according to Fisher´s least significant difference test (*p* ≤ 0.05). DW: dry weight.

**Table 4:** ABTS antioxidant capacity in tomato leaves from plants treated with Se and Cu NPs.

Se NPs (mg L^−1^)	Cu NPs (mg L^−1^)			
	0	10	50	250
**NS ABTS hidrophilic (µmol g^−1^ DW)**				
*** 0	* 73.49 ± 0.4 g	74.92 ± 0.9 fg	74.96 ± 1.1 fg	75.76 ± 1.0 efg
**1**	77.16 ± 1.2 bcdefg	77.07 ± 1.0 cdefg	78.46 ± 1.4 bcdef	76.15 ± 1.4 defg
**10**	81.16 ± 1.9 ab	78.39 ± 1.4 bcdef	79.91 ± 0.9 abcd	79.03 ± 1.6 bcde
**20**	79.30 ± 1.2 bcde	76.04 ± 2.9 defg	80.72 ± 1.7 abc	83.96 ± 1.4 a
** ABTS lipophilic (µmol g^−1^ DW)				
*** 0	** 54.86 ± 0.3 c	56.12 ± 0.1 bc	56.25 ± 0.4 bc	57.79 ± 0.4 ab
**1**	55.74 ± 0.4 bc	56.47 ± 0.5 bc	56.58 ± 0.4 bc	56.47 ± 06 bc
**10**	48.75 ± 0.5 d	51.15 ± 1.2 d	54.36 ± 2.2 c	50.82 ± 1.1 d
**20**	55.33 ± 0.8 bc	56.14 ± 0.4 bc	55.09 ± 0.8 c	59.79 ± 1.8 a
** Total antioxidant capacity (µmol g^−1^ DW)				
*** 0	** 128.35 ± 0.36 f	131.04 ± 0.84 cdef	131.21 ± 1.15 bcdef	133.54 ± 1.29 bcde
**1**	132.90 ± 1.01 bcdef	133.54 ± 1.22 bcde	135.04 ± 1.09 bc	132.62 ± 1.17 bcdef
**10**	129.91 ± 1.82 def	129.54 ± 1.93 ef	134.27 ± 2.21 bcd	129.85 ± 1.47 def
**20**	134.62 ± 1.47 bc	132.18 ± 2.96 bcdef	135.81 ± 2.35 b	143.75 ± 2.22 a

ANOVA significance for both main factors and interactions are ^NS^ (not significant), * <0.05, ** <0.01, *** <0.001. Means ± standard error between rows and columns with a common letter are not significantly different according to Fisher´s Least Significant Difference test (*p* ≤ 0.05). NPs: nanoparticles; ABTS: 2,2′-azino-bis(3-ethylbenzthiazolin-6-sulfonic acid.

**Table 5:** Enzymatic activity in tomato leaves of plants treated with Se and Cu NPs.

Se NPs (mg L^−1^)	Cu NPs (mg L^−1^)			
	0	10	50	250
**^NS^** **Ascorbate Peroxidase** **(APX)** **(U g^–1^ TP)**				
* 0	* 176.20 ± 58.5 b	248.77 ± 109.4 ab	134.08 ± 27.7 b	236.40 ± 105.2 b
**1**	139.16 ± 30.6 b	129.19 ± 36.9 b	268.43 ± 32.74 ab	134.45 ± 16.6 b
**10**	220.99 ± 53.8 b	206.99 ± 61.6 b	260.37 ± 55.5 ab	223.70 ± 76.4 b
**20**	164.27 ± 67.9 b	338.07 ± 56.1 ab	455.61 ± 184.5 a	264.69 ± 56.8 ab
**^NS^** **Glutathione Peroxidase (GPX) (U g^–1^ TP)**				
* 0	* 29.92 ± 8.9 c	115.61 ± 30.5 bc	289.25 ± 32.6 bc	206.27 ± 28.0 bc
**1**	373.60 ± 192.3 abc	546.45 ± 219.7 ab	821.40 ± 543.4 a	196.05 ± 33.2 bc
**10**	201.45 ± 33.4 bc	221.61 ± 49.3 bc	423.62 ± 106.0 abc	223.20 ± 103.4 bc
**20**	121.76 ± 33.4 bc	281.83 ± 78.1 bc	349.54 ± 81.4 bc	403.73 ± 158.6 abc
**^NS^** **Catalase (U g^–1^ TP)**				
**^NS^** **0**	^NS^ 815.35 ± 136.3 a	780.40 ± 165.1 a	783.45 ± 279.0 a	597.42 ± 146.2 a
**1**	1025.78 ± 277.8 a	651.63 ± 123.7 a	892.28 ± 226.0 a	701.27 ± 145.5 a
**10**	1100.92 ± 241.6 a	744.87 ± 162.3 a	777.65 ± 185.4 a	736.32 ± 285.1 a
**20**	747.50 ± 190.7 a	661.97 ± 167.7 a	995.52 ± 120.2 a	739.78 ± 160.7 a
**^NS^** **Superoxide Dismutase (SOD) (U mL^−1^)**				
**^NS^** **0**	* 110.01 ± 26.6 bc	139.23 ± 21.7 abc	134.97 ± 34.6 abc	76.50 ± 16.0 c
**1**	179.44 ± 27.1 a	89.98 ± 20.3 bc	143.80 ± 29.4 abc	122.96 ± 16.5 abc
**10**	91.49 ± 17.0 bc	99.36 ± 25.2 bc	123.02 ± 21.1 abc	107.47 ± 31.3 bc
**20**	97.14 ± 27.1 bc	137.88 ± 32.3 abc	147.55 ± 16.2 ab	124.82 ± 12.1 abc
* Phenylalanine Ammonia Liasa (PAL) (U g^−1^ TP)				
* 0	** 4.79 ± 0.7 c	5.37 ± 1.5 c	11.81 ± 4.2 ab	6.52 ± 1.4 c
**1**	8.13 ± 1.2 bc	14.51 ± 2.9 a	8.16 ± 0.5 bc	8.10 ± 0.9 bc
**10**	6.98 ± 0.6 c	7.71 ± 1.1 bc	7.15 ± 0.8 c	6.14 ± 0.7 c
**20**	5.83 ± 1.0 c	7.57 ± 0.7 bc	7.81 ± 0.7 bc	6.60 ± 0.9 c

ANOVA significance for both main factors and interactions are ^NS^ (not significant), * <0.05, ** <0.01, *** <0.001. Means ± standard error between rows and columns with a common letter are not significantly different according to Fisher´s least significant difference test (*p* ≤ 0.05). TP: total proteins.

**Table 6:** Non-enzymatic antioxidant compounds in tomato fruits obtained from plants treated with Se and Cu NPs.

Se NPs (mg L^−1^)	Cu NPs (mg L^−1^)			
	0	10	50	250
** Vitamin C (mg 100 g^−1^ FW)				
* 0	** 12.76 ± 0.5 bcde	12.03 ± 0.5 cde	15.11 ± 0.8 ab	13.64 ± 0.7 bcd
**1**	14.81 ± 1.4 abc	14.52 ± 1.6 abc	17.31 ± 1.1 a	10.41 ± 2.4 e
**10**	11.00 ± 0.7 de	14.67 ± 0.8 abc	14.96 ± 0.5 ab	13.79 ± 0.6 bcd
**20**	11.59 ± 0.5 de	12.91 ± 1.1 bcde	11.59 ± 0.4 de	11.44 ± 0.9 de
**^NS^ Glutathione (mg 100 g^−1^ DW)**				
**^NS^** **0**	* 3.92 ± 0.4 cd	4.66 ± 0.4 abc	4.47 ± 0.2 bcd	4.64 ± 0.2 abc
**1**	4.71 ± 0.3 abc	4.92 ± 0.4 ab	4.80 ± 0.3 abc	4.53 ± 0.2 abcd
**10**	4.91 ± 0.4 ab	4.29 ± 0.4 bcd	4.88 ± 0.1 ab	5.41 ± 0.4 a
**20**	3.72 ± 0.2 d	4.48 ± 0.2 bcd	5.14 ± 0.4 ab	4.47 ± 0.4 bcd
** Flavonoids (mg 100 g^−1^ DW)				
*** 0	* 79.60 ± 4.2 de	96.23 ± 9.6 abc	71.12 ± 9.0 e	79.18 ± 6.0 de
**1**	88.00 ± 1.3 abcd	89.16 ± 6.6 abcd	85.42 ± 3.7 bcde	85.67 ± 3.3 bcde
**10**	74.86 ± 5.0 de	74.69 ± 4.2 de	69.62 ± 6.0 e	74.03 ± 6.8 de
**20**	100.63 ± 6.0 ab	102.63 ± 5.9 a	79.27 ± 4.4 de	81.35 ± 5.5 cde
**^NS^ Phenols (mg 100 g^−1^ DW)**				
** 0	* 15.02 ± 0.6 abc	15.54 ± 0.8 ab	13.50 ± 1.1 bc	16.17 ± 0.4 a
**1**	12.88 ± 0.8 c	13.64 ± 0.5 bc	13.87 ± 1.2 bc	13.43 ± 0.5 bc
**10**	13.70 ± 0.3 bc	14.51 ± 0.7 abc	13.57 ± 0.6 bc	14.64 ± 0.9 abc
**20**	15.41 ± 0.9 ab	14.84 ± 0.6 abc	15.22 ± 0.8 ab	16.36 ± 1.2 a
**^NS^ Lycopene (mg 100 g^−1^ DW)**				
*** 0	* 54.91 ± 3.9 abc	33.76 ± 7.8 ef	38.43 ± 9.4 def	38.21 ± 8.8 def
**1**	52.87 ± 6.1 abcd	58.69 ± 4.6 a	59.81 ± 3.4 a	52.02 ± 1.7 abcd
**10**	45.46 ± 5.8 abcde	27.98 ± 5.7 f	40.66 ± 2.9 bcdef	39.78 ± 5.8 bcdef
**20**	38.80 ± 7.1 cdef	60.91 ± 2.0 a	55.60 ± 4.9 ab	47.73 ± 5.3 abcde
**^NS^ β-carotene (mg 100 g^−1^ DW)**				
**^NS^ 0**	* 8.05 ± 1.8 ab	7.93 ± 1.1 ab	7.70 ± 1.5 ab	15.25 ± 7.2 a
**1**	12.13 ± 2.8 ab	12.89 ± 2.7 ab	7.76 ± 2.5 ab	5.93 ± 2.2 b
**10**	8.04 ± 1.3 ab	10.18 ± 2.5 ab	7.55 ± 1.9 b	10.00 ± 2.3 ab
**20**	6.84 ± 2.5 b	7.90 ± 1.7 ab	6.36 ± 1.9 b	5.72 ± 2.0 b

ANOVA significance for both main factors and interactions are ^NS^ (not significant), * <0.05, ** <0.01, *** <0.001. Means ± standard error between rows and columns with a common letter are not significantly different according to Fisher´s least significant difference test (*p* ≤ 0.05).

**Table 7:** Parameters of tomato fruit quality obtained from plants treated with Se and Cu NPs.

Se NPs (mg L^−1^)	Cu NPs (mg L^−1^)			
	0	10	50	250
**^NS^ Firmness (kg cm^−2^)**				
**^NS^ 0**	* 4.65 ± 0.8 bc	4.85 ± 0.7 bc	5.13 ± 1.0 abc	6.07 ± 0.9 abc
**1**	4.88 ± 0.7 bc	5.88 ± 0.9 abc	4.92 ± 0.5 bc	5.15 ± 0.4 abc
**10**	6.27 ± 0.9 ab	5.62 ± 0.4 abc	6.87 ± 0.7 a	5.55 ± 0.4 abc
**20**	5.00 ± 0.7 abc	5.05 ± 0.4 abc	5.93 ± 0.7 abc	4.18 ± 0.3 c
**^NS^ pH**				
**^NS^ 0**	* 4.26 ± 0.03 ab	4.22 ± 0.03 ab	4.23 ± 0.05 ab	4.29 ± 0.08 a
**1**	4.20 ± 0.02 ab	4.16 ± 0.03 b	4.23 ± 0.01 ab	4.24 ± 0.04 ab
**10**	4.21 ± 0.02 ab	4.22 ± 0.04 ab	4.30 ± 0.03 a	4.25 ± 0.02 ab
**20**	4.22 ± 0.02 ab	4.25 ± 0.04 ab	4.20 ± 0.04 ab	4.23 ± 0.04 ab
**^NS^ Electric conductivity (mS cm^−1^)**				
**^NS^ 0**	* 3.40 ± 0.23 abc	3.05 ± 0.35 abc	2.87 ± 0.15 bc	3.45 ± 0.10 ab
**1**	2.73 ± 0.31 c	3.57 ± 0.30 ab	2.98 ± 0.21 abc	3.38 ± 0.23 abc
**10**	3.47 ± 0.16 ab	3.62 ± 0.25 a	3.43 ± 0.27 abc	3.08 ± 0.37 abc
**20**	3.23 ± 0.26 abc	3.67 ± 0.27 a	3.12 ± 0.25 abc	3.68 ± 0.15 a
**^NS^ Total Soluble Solids (ºBrix)**				
**^NS^ 0**	* 4.83 ± 0.17 b	5.17 ± 0.17 ab	4.83 ± 0.17 b	5.00 ± 0.01 ab
**1**	5.00 ± 0.01 ab	5.00 ± 0.01 ab	5.00 ± 0.01 ab	5.00 ± 0.01 ab
**10**	4.83 ± 0.17 b	5.00 ± 0.01 ab	5.00 ± 0.01 ab	4.83 ± 0.17 b
**20**	5.00 ± 0.01 ab	5.00 ± 0.01 ab	5.00 ± 0.01 ab	5.33 ± 0.33 a
* Titratable Acidity (% citric acid)				
* 0	* 0.39 ± 0.02 de	0.39 ± 0.01 de	0.42 ± 0.01 bcd	0.43 ± 0.01 abcd
**1**	0.42 ± 0.01 bcd	0.42 ± 0.02 bcd	0.42 ± 0.02 bcd	0.44 ± 0.02 abc
**10**	0.36 ± 0.03 e	0.42 ± 0.02 cd	0.42 ± 0.02 bcd	0.47 ± 0.02 a
**20**	0.45 ± 0.01 abc	0.47 ± 0.02 a	0.47 ± 0.02 a	0.41 ± 0.01 cde

ANOVA significance for both main factors and interactions are ^NS^ (not significant), * <0.05, ** <0.01, *** <0.001. Means ± standard error between rows and columns with a common letter are not significantly different according to Fisher´s least significant difference test (*p* ≤ 0.05).
